# Evaluation and Prevention of Perioperative Respiratory Failure

**DOI:** 10.3390/jcm13175083

**Published:** 2024-08-27

**Authors:** Jacqueline Palermo, Spencer Tingey, Ashish K. Khanna, Scott Segal

**Affiliations:** 1 Wake Forest University School of Medicine, Winston-Salem, NC 27101, USA; japalerm@wakehealth.edu (J.P.); stingey@wakehealth.edu (S.T.); 2 Department of Anesthesiology, Section on Critical Care Medicine, Atrium Health Wake Forest Baptist Medical Center, Wake Forest University School of Medicine, Winston-Salem, NC 27157, USA; akhanna@wakehealth.edu; 3 Department of Anesthesiology, Atrium Health Wake Forest Baptist Medical Center, Wake Forest University School of Medicine, Winston-Salem, NC 27157, USA

**Keywords:** respiratory failure, postoperative, prevention, monitoring, evaluation

## Abstract

Respiratory failure is a common perioperative complication. The risk of respiratory failure can be reduced with effective preoperative evaluation, preventative measures, and knowledge of evidence-based management techniques. Effective preoperative screening methods include ARISCAT scoring, OSA screening, and the LAS VEGAS score (including the ASA physical status score). Evaluation by the six-minute walk test and a routine pulmonary physical exam has been shown to be effective at predicting postoperative pulmonary complications, whereas evidence on the predictive power of pulmonary function tests and chest radiography has been inconclusive. Preoperative smoking cessation and lung expansion maneuvers have been shown to decrease the risk of pulmonary complications postoperatively. Intraoperative management techniques that decrease the pulmonary complication risk include neuromuscular blockade reversal with sugammadex, limiting surgical times to less than 3 h when possible, lung-protective ventilation techniques, and multimodal analgesia to decrease opioid usage. In the immediate postoperative period, providers should be prepared to quickly treat bronchospasm, hypoventilation, and upper airway obstruction. For post-surgical patients who remain in the hospital, the risk of pulmonary complications can be decreased with lung expansion techniques, adequate analgesia, automated continuous postoperative ward monitoring, non-invasive ventilatory support, and early mobilization. This article was written to analyze the available literature on this topic in order to learn and practice the prevention of perioperative respiratory failure when caring for patients on a daily basis.

## 1. Introduction

The number of surgical procedures performed globally continues to increase over time. From 2005 to 2013, there was a 33.6% increase in procedures worldwide, with a total of 312.9 million in 2012 [1]. For each surgical patient, the possibility of perioperative complications must be considered and planned for. Cardiothoracic patients have a 40% incidence of respiratory failure following surgical procedures [2]. In all surgical patients, the rate of pulmonary complications has been reported to be 5% to 10% [3]. Postoperative pulmonary complications consist of respiratory system problems after surgery such as prolonged usage of oxygen therapy, atelectasis, pneumonia, bronchitis, or respiratory failure [4]. Respiratory failure can lead to prolonged hospital stays of up to two weeks, increased postoperative mortality, ICU admission, and increased hospital cost [2]. It is important to understand how to screen, evaluate, and manage patients with respiratory failure during the perioperative timeframe as postoperative mortality accounts for 7.7% of deaths globally [5].

Respiratory failure occurs when the respiratory system is either unable to provide oxygen to the body, causing hypoxemia, or unable to remove carbon dioxide, causing hypercapnia, or both, leading to worsened prognosis and outcomes. McAlister et al. noted five significant predictors of pulmonary complications from 272 patients undergoing nonthoracic procedures: hypercapnia of ≥45 mmHg, a maximal laryngeal height of ≤4 cm, a forced expiratory time of ≥9 s, smoking of ≥40 pack-years, and a body mass index of ≥30 [6]. These risk factors are common, so it is essential to have a prevention strategy for this perioperative complication. There are four types of respiratory failure: Type 1 (hypoxemic), Type 2 (hypercapnic), Type 3 (perioperative), and Type 4 (shock). This review will focus on Type 3, perioperative respiratory failure, covering screening, evaluation, and management throughout patients’ hospital stays.

## 2. Preoperative Period

### 2.1. Screening Tests

Effective preoperative screening is essential and should include an appropriate pulmonary evaluation, with the goal of limiting pulmonary complications and improving outcomes in the preoperative, intraoperative, and postoperative periods. One screening method performed preoperatively is the Assess Respiratory Risk in Surgical Patients in Catalonia (ARISCAT) risk score [7]. This risk score (Table 1) classifies patients into three levels, high, intermediate, and low, based on the following patient- and procedure-related risks: preoperative oxygen saturation, advanced age, respiratory infection within the past month, preoperative anemia, surgical procedure lasting more than two hours, and upper abdominal or thoracic surgical incision. A prospective cohort of 2400 patients showed an increased ARISCAT score correlated with an increased rate of postoperative pulmonary complications [8]. Another screening method is the Gupta Perioperative Risk for Myocardial Infarction or Cardiac Arrest score, which takes into account age, functional status, ASA class, creatinine levels, and type of procedure [9].

Undiagnosed obstructive sleep apnea (OSA) remains alarmingly common in the perioperative period (as many as 80% of patients with clinically significant disease are unaware), and it can lead to perioperative atelectasis, particularly in obese patients, by reducing lung volumes, specifically functional residual capacity. Patients with OSA and sleep-disordered breathing (SDB) are at an increased risk of postoperative pulmonary and cardiovascular events [10]. For example, SDB is one of the major drivers of opioid-induced respiratory depression (OIRD) in the post-surgical and medical population [11,12]. Polysomnography is recommended to confirm the diagnosis as a gold standard for obstructive sleep apnea. An easy-to-use screening method, the STOP-BANG score, is commonly employed to screen for OSA. The STOP-BANG score described in Chung et. al. asks patients about snoring, tiredness, observed apnea, pressure (high or low blood pressure), body mass index > 35 kg/m^2^, age older than 50 years, neck circumference > 40 cm, and the male gender [13]. One point is allotted for each item and a resulting score > 3 is indicative of being at risk for OSA. If the resulting score is greater than 5, cardiologic and pneumological evaluation should be considered [14]. An increase in this score does not predict postoperative hypoxemia [15]. In general, STOP-BANG (Table 2) is sensitive and therefore a powerful screening tool, but it is not specific, and many false positives are seen [16].

If patients have diagnosed and treated OSA, it is important to continue regular positive airway pressure (PAP) therapy, limit respiratory depression by using multimodal analgesia, avoid supine positioning postoperatively by raising the head of the bed, and judiciously administer fluids [17].

The Obesity Surgery Mortality Risk Score is also used to determine postoperative mortality in bariatric surgery, but it has failed to demonstrate an association with rates of complications in a prospective study of 321 patients undergoing bariatric surgery [18]. 

Another proposed screening test for predicting postoperative pulmonary complications is the Local Assessment of Ventilatory management during General Anesthesia for Surgery (LAS VEGAS), which employs baseline patient characteristics including functional status and the American Society of Anesthesiologists’ (ASA) physical status score, as well as intraoperative ventilator settings and vital signs, as predictors for pulmonary complications in the first five postoperative days. This 6063-patient international prospective study’s primary outcome was a postoperative pulmonary complication (PPC) including respiratory failure within five days of surgical procedures. This score risk has a moderate ability to predict PPC [19]. 

The ASA physical status score (typically “ASA score”) assesses a patient’s pre-anesthesia medical comorbidities:

1. normal, healthy patient;
2. patient with mild systemic disease;
3. patient with severe systemic disease;
4. patient with severe systemic disease that is a constant threat to life;
5. patient not expected to survive without the operation;
6. patient is brain-dead and their organs are being removed for donor purposes.

A meta-analysis of twelve studies demonstrated a positive association between higher ASA class and greater risk for PPC [20]. 

The American College of Physicians’ (ACP) recommendations to reduce perioperative pulmonary complications include the evaluation of the pulmonary risk of chronic obstructive pulmonary disease (COPD) for all patients greater than 60 years old, ASA class II or greater, and with congestive heart failure [21]. The ACP also recommends caution in patients having prolonged surgical procedures (>3 h), thoracic surgery, abdominal surgery, neurosurgery, vascular surgery, and general anesthesia. Patients undergoing lung surgery are recommended to undergo nutritional screening, to undertake pre-habilitation, and to avoid preoperative sedatives [22]. Another recommendation is the measurement of serum albumin, as lower values (<35 gL^−1^) increase the risk of pulmonary complication [23]. Patients with high postoperative pulmonary complication risk should receive deep breathing exercises. Preoperative spirometry and chest radiography are not recommended to predict the risk of pulmonary complications. A 2022 meta-analysis of 46 studies that used pulmonary function test findings as index tests and postoperative pulmonary complications as a target condition revealed inconclusive study findings: 65% argued for and 35% argued against preoperative spirometry [24].

### 2.2. Evaluation

Preoperative respiratory functional evaluation may help predict the risk of PPC. Tests that have been demonstrated to be effective include the six-minute walk test, lung function tests, clinical and radiographic evaluations for respiratory infection, and sometimes testing for COPD through chest radiography [19,20,21,22,24,25]. 

Cardiopulmonary exercise testing (CPET) is the generally accepted standard for assessing functional capacity, but it is resource-intensive and is not widely available [26]. A 2018 systematic literature review provided the following as recommended indications for CPET:

(1) To estimate the likelihood of perioperative morbidity and mortality and contribute to preoperative risk assessment.
(2) To inform the processes of multidisciplinary shared decision-making and consent.
(3) To guide clinical decisions about the most appropriate level of perioperative care (ward vs. critical care).
(4) To direct preoperative referrals/interventions to optimize comorbidities.
(5) To identify previously unsuspected pathology.
(6) To evaluate the effects of neoadjuvant cancer therapies, including chemotherapy and radiotherapy.
(7) To guide prehabilitation and rehabilitation training programs.
(8) To guide intraoperative anesthetic practice [27].

The six-minute walk test (6-MWT) is a simpler and still-effective alternative to CPET that measures how far patients can walk along a 30 m flat route, turning 180° every 30 m, in 6 min. A prospective evaluation of 117 patients undergoing major surgical procedures including thoracotomy, sternotomy, or upper abdominal laparotomy found that the 6-MWT was a useful predictor for postoperative complications and hospital length of stay (*p* < 0.0001; [25]). 

Pulmonary function tests (PFTs) are often used preoperatively for high-risk surgical procedures (lung resections) or if patients experience respiratory symptoms. PFTs include spirometry, body plethysmography, gas diffusion, and cardiopulmonary exercise tests. In a series of scoliosis patients, Zhang et al. found that there was an increased incidence of postoperative pulmonary complications when PFTs indicated abnormal function, particularly when the forced vital capacity was reduced [28]. Correlations with reduced DL_CO_ and/or FEV_1_ have been reported in other types of surgical procedures, including liver transplant, esophagectomy, lung cancer, and gastric resection. However, a systematic review of 46 studies found inconclusive evidence for any valuable role of PFTs in predicting adverse outcomes, except in upper abdominal surgical procedures [24]. Thus, the role of this testing appears to be confined to specific surgical populations.

Medical history, including a history of respiratory infection, and physical examination, including auscultation of breath sounds and thoracic percussion, are often part of a routine preoperative evaluation. Kocabas et al. prospectively tested 60 patients’ respiratory status the day before their surgical procedure and 15 days after [29]. They found more pulmonary complications when there were wheezes, rhonchi, prolonged expiratory phase, dullness to percussion, and decreased breath sounds observed preoperatively. Similarly, in a retrospective review of 2291 patients undergoing elective abdominal procedures, abnormal findings on lung examination predicted PPC (OR 5.8), whereas spirometry findings did not [30].

Chest radiographs are sometimes used, but a meta-analysis of 21 reports found that only 0.1% had findings sufficient to change the patients’ course and management [31]. They also noted the clinical information provided from radiographs does not justify the cost of the test, so it is rarely used. A 2023 American College of Radiology Appropriateness Criteria statement on routine chest imaging concluded, “Routine preoperative chest imaging is usually not appropriate in patients without cardiopulmonary disease undergoing noncardiothoracic surgery” [32]. 

Arterial blood gas measurement is the standard for the assessment of oxygenation, ventilation, and acid–base status. This remains an effective evaluation tool during procedures requiring arterial line placement but otherwise has mainly been replaced by non-invasive monitoring [33]. 

The future direction of perioperative pulmonary evaluation can improve the prediction of PPCs with use of the six-minute walk test and with early identification of respiratory symptoms and signs during preoperative physical examinations.

### 2.3. Management

**A.**  **Preoperative Considerations and Specific Populations**

Preoperative management for patients can include patient education regarding smoking cessation and lung expansion maneuvers for high-risk patients. 

A meta-analysis of 107 studies found that preoperative smoking was associated with an increased risk of postoperative complications, including respiratory failure, infection, and admission to the intensive care unit [34]. A retrospective study of 500 patients undergoing coronary artery bypass found that patients who stopped smoking for eight weeks had a threefold decrease in the incidence of PPC [35]. Similarly, a meta-analysis of 24 studies in shoulder arthroplasty found improved outcomes from smoking cessation for at least one month preoperatively [36]. However, it may be necessary to achieve smoking cessation for at least several weeks (>4) for the intervention to reduce PPCs; a meta-analysis of 25 studies found no evidence of reduction in complications with cessation < 2 or 2–4 weeks preoperatively [37]. Nonetheless, the perioperative period is often characterized as a “teachable moment” and thus as an opportunity to attempt smoking cessation, irrespective of the time before the surgical procedure, to achieve the well-known benefits of quitting. Evidence suggests perioperative quitting leads to longer-term cessation in a sizeable fraction of patients [38]. 

Lung expansion techniques, including active breathing, forced expiration, and incentive spirometry, may also lessen PPC. Mans et al. performed a systematic review of eight randomized and quasi-randomized trials of 295 participants [39]. They found preoperative muscle training improved respiratory function postoperatively and significantly decreased the risk of PPC (relative risk 0.48, 95% CI 0.26 to 0.89). 

Special considerations should be made for specific populations with higher-than-usual risk of PPC: patients with severe OSA, COPD, asthma, interstitial lung disease, cancer, pulmonary hypertension, malnourishment, and chronically ventilated patients. Screening tests such as STOP-BANG and ARISCAT may help with risk stratification in these higher-risk populations. Patients with OSA or sleep-disordered breathing have been shown to have an increased odds ratio for emergent intubation and mechanical ventilation, non-invasive ventilation, and atrial fibrillation [40]. When OSA is diagnosed or suspected, perioperative non-invasive ventilation may be indicated.

**B.**  **Intraoperative Management**

Intraoperative techniques for the prevention of perioperative respiratory failure should be considered in the context of the surgical procedure. 

Neuromuscular-blocking (NMB) drugs facilitate major surgical procedures, particularly intracavitary procedures. However, residual blockade can cause hypoventilation, diaphragmatic dysfunction, and atelectasis, and it may increase postoperative pulmonary complications. Contemporary data suggest that residual NMB can complicate any anesthetic utilizing NMB drugs in up to 70% of cases, and the American Society of Anesthesiologists has recommended the use of quantitative (vs. traditional qualitative) NMB monitoring to minimize this complication [41]. In addition, the emergence of sugammadex for NMB reversal has been shown to significantly reduce the risk of PPC, albeit at a somewhat higher cost than neostigmine. A meta-analysis of 43 trials studying 4839 subjects demonstrated a reduced incidence of pneumonia, hospital length of stay, improved patient-reported quality of recovery, and overall mortality with sugammadex [42].

Surgical procedure length is a risk factor for PPC. Duggan et al. recommended that procedures last less than three hours when possible [2]. Meta-analyses of operations of various types have confirmed the effect of longer surgical duration [20,43]. Upper abdominal surgical procedures and both upper and lower abdominal incisions have been shown to be risk factors for PPC [44]. Clearly, these risk factors are not always amenable to interventions to reduce them, so it is reasonable to accentuate various other protective strategies in these higher-risk procedures.

During general anesthesia, there can be decreased functional residual capacity, which can lead to atelectasis, but volutrauma and barotrauma complicate the use of larger tidal volumes. Lung-protective ventilation techniques, borrowed from ICU strategies to minimize complications in ARDS, will ideally reduce alveolar overdistention and atelectasis. The components of lung-protective ventilation include a low tidal volume, a positive end-expiratory pressure (PEEP), and recruitment maneuvers to reopen the alveoli [45]. A meta-analysis of 63 trials found that lung-protective ventilation reduces the risk of pulmonary complications and the need for postoperative mechanical ventilation [46]. Another meta-analysis of 19 randomized trials found that, when comparing low tidal volume (6–8 mL/kg IBW) with high tidal volume (≥10 mL/kg IBW) in patients without lung disease, there was a decrease in postoperative pneumonia and postoperative invasive ventilation when using low tidal volumes [47]. 

The use of regional anesthesia versus general anesthesia should be considered. Hausman et al. reviewed data from the National Surgical Quality Improvement Program (NSQIP) and compared a cohort of 2644 patients who received general anesthesia against a propensity score-matched cohort of 2644 who received regional anesthesia [48]. They found that patients with COPD who had regional instead of general anesthesia had a decreased incidence of pneumonia, morbidity, ventilator dependence, and postoperative intubation. Conversely, a systematic review involving 10,488 patients comparing spinal, epidural, and general anesthesia for total hip or knee arthroplasties found equal morbidity and perioperative outcomes [49]. A more recent large, randomized trial of spinal vs. general anesthesia for hip fracture repair found no difference in outcomes, as did a meta-analysis of several earlier trials [50,51]. These conflicting results could be due to differences in patient risk factors, the type of surgical procedure, or postoperative care. Nonetheless, it would be reasonable to consider regional techniques in appropriate procedures for high-risk patients.

Multimodal analgesia, in which agents from several drug classes such as nonsteroidal anti-inflammatory drugs (NSAIDs), acetaminophen, gabapentinoids (gabapentin and pregabalin), and opioids are combined to reduce opioid use and enhance analgesia, has been suggested as a technique to reduce PPC. Early work clearly demonstrated the benefit of epidural analgesia for postoperative analgesia, compared to conventional opioids, but questioned the efficacy of other agents [52]. More recent trials of analgesic peripheral nerve blocks have confirmed efficacy in certain surgical populations, such as those who have had joint replacements, thoracic resections, and cardiac procedures. Multimodal analgesia may indeed reduce opioid consumption, but the effect on PPC remains less clear [53]. Moreover, some evidence suggests that gabapentinoids may actually be associated with increased PPC, while NSAIDs and acetaminophen may reduce them [54].

**C.**  **Postoperative Care**

In the immediate postoperative period, respiratory complications can occur as the patient completes their emergence from anesthesia but remains susceptible to anesthetic drugs and the mechanical effects of endotracheal intubation or to the compromise of the airway. Providers should be prepared for rapid resolution of respiratory compromise in the Post Anesthesia Care Unit (PACU), including bronchospasm, hypoventilation, and upper airway obstruction.

(i). Bronchospasm

Bronchospasm is a common intraoperative event but a less common PACU complication, affecting approximately 0.3% of patients in one large retrospective series [55]. Etiologies include but are not limited to an allergic response to medication, medication-incited histamine release, aspiration, and exacerbation of underlying asthma or COPD. Risk factors include older age and pre-existing COPD. Bronchospasm can also occur as a reflexive response to tracheal stimulation, especially as the inhalational anesthetic wears off and is no longer exerting bronchodilatory effects [56]. Clinical presentation can include dyspnea or tachypnea, wheezing, the sensation of chest tightness, low tidal volume, prolonged expiration, and hypercapnia. Bronchodilators, usually a short-acting beta-2-agonist such as albuterol, are first-line pharmacotherapy. In more severe cases of bronchospasm, the addition of ipratropium bromide, a short-acting anticholinergic agent, may be appropriate. Systemic glucocorticoids may be indicated for persistent or recurrent bronchospasm. Some maneuvers to help attenuate the risk of bronchospasm in high-risk patients include extubation under a deep plane of anesthesia before the return of airway reflexes and the use of dexmedetomidine near the end of anesthesia [57,58].

(ii). Hypoventilation

Hypoventilation can occur due to the residual effects of anesthesia, especially in cases of incomplete reversal of neuromuscular-blocking (NMB) agents or of heavy opioid use. Monitoring for complete reversal of NMB is particularly important in patients with underlying lung disease, as hypoventilation may increase the risk of PPC [59]. General anesthetics can have synergistic effects with opioids and benzodiazepines and contribute to oversedation in the PACU [60].

(iii). Acute upper airway obstruction

Acute upper airway obstruction is most common immediately following surgical procedures, often due to the physical effects of intubation on the pharynx and tongue. Obstruction can be caused by the accumulation of secretions in the pharynx, the tongue obstructing the airway due to edema or posterior movement, laryngeal edema, vocal cord paralysis, and laryngospasm [56]. Laryngospasm following endotracheal tube removal accounts for 23% of critical postoperative respiratory events in adults [61].

Clinical presentation can include stridor or aphonia depending on whether the airway is completely obstructed. Other signs of an obstruction include dyspnea, tachypnea, tachycardia, and diaphoresis.

Airway obstruction requires emergent management. The application of continuous positive airway pressure, non-invasive ventilation, changes in head position, jaw thrust and/or chin lift, oropharyngeal suctioning, and placement of a oropharyngeal or nasopharyngeal airway are maneuvers that can be quickly applied [62]. In some cases, the use of a short-acting neuromuscular-blocking agent or of immediate intubation is required. In cases of an incomplete obstruction, bronchodilation or the inhalation of a mixture of helium and oxygen may be beneficial [63].

### 2.4. Prevention of Postoperative Respiratory Failure on Hospital Wards 

Following PACU care, post-surgical patients who remain in the hospital continue to be at risk of respiratory failure. Proper preventative measures, including appropriate monitoring, adequate ventilation support, lung expansion maneuvers, and adequate pain control, are important to decrease the risk of pulmonary complications.

Appropriate monitoring of postoperative patients is the first step in preventing complications. Pulse oximetry is commonly used, and experts advocate for increasing the use of capnography, at least in patients receiving opioids. A multimodal monitoring approach comprising monitoring of respiratory rate, tidal volume, O_2_ saturation, and ETCO_2_ can provide more effective monitoring than any of these commonly used methods when used as a single measure. Bedside monitors that are portable, continuous, and have an effective central alarm and a noise filter should be used [64]. Remote monitoring should be used in conjunction with frequent direct observation of patients.

Lack of lung expansion is common postoperatively due to a shift from normal breathing to an increase in shallow breathing. Pain associated with deep breaths or coughs, as well as prolonged recumbent positions and decreased mucociliary clearance, can cause this shift in breathing pattern [65]. Lung expansion techniques may help reduce the rates of respiratory complications. For example, a clinical trial of 456 patients undergoing an abdominal procedure concluded that the most efficient regimen is deep breathing exercises for low-risk patients and incentive spirometry for high-risk patients [66]. However, a 2014 meta-analysis of 12 randomized trials found no overall benefit of incentive spirometry [67].

Adequate analgesia can also facilitate patients’ return to a normal breathing pattern and allows for improved lung expansion, as well as enabling earlier ambulation. Traditionally, parenteral opioids have been used for postoperative pain control. The PRODIGY study revealed that up to 46% of continuously monitored hospital ward patients on parenteral opioids had evidence of OIRD [12]. In addition, commonly used agents such as sedatives further increase the risk of adverse outcomes in patients receiving postoperative opioids. However, the use of epidural analgesia and intercostal nerve blocks should be considered for pain control due to their effectiveness in decreasing respiratory complications [68,69].

Automated continuous postoperative ward monitoring may be able to improve outcomes over current intermittent monitoring practices by detecting changes in vital signs occurring before acute cardiorespiratory events [64]. Subtle changes in vital signs can occur at least eight to twelve hours before an acute event [70]. These changes in vital signs can be difficult to recognize with intermittent monitoring. The use of an effective set of devices that are portable and preferably wearable for continuous monitoring would allow for timely therapies that could decrease the incidence of acute respiratory failure events [71].

Non-invasive ventilatory support methods may be considered in patients who develop respiratory insufficiency. Effective ventilatory support options to consider for the treatment of respiratory failure include continuous positive airway pressure (CPAP) and high-flow nasal cannula oxygen. A 2014 meta-analysis of 10 studies (albeit characterized as of very low quality by the reviewers) found a decrease in atelectasis and pulmonary complications [72]. However, a recent large prospective trial of CPAP vs. usual care found no reduction in PPC with CPAP [73].

Early mobilization after a surgical procedure is an important part of preventing respiratory failure and other PPCs during recovery from the procedure. In a cohort study of 8653 patients, each 4 min increase in mobilization per monitored hour was associated with a 25% reduction in the odds of composite complications and with shortened hospital length of stay [74].

## 3. Conclusions

Perioperative respiratory failure remains common and is associated with significant clinical and health care resource implications. Proactive screening, evaluation, and prevention are of critical importance to improve outcomes. 

## Figures and Tables

**Table 1:** Assess Respiratory Risk in Surgical Patients in Catalonia (ARISCAT) risk score [7].

Score Components		Risk Score
Age	≤50 years	0
	51–80 years	3
	>80 years	16
Preoperative oxygen saturation	≥96%	0
	91–95%	8
	≤90%	24
Respiratory infection in past 1 month	No	0
	Yes	17
Preoperative hemoglobin < 10 g/dL	No	0
	Yes	11
Incision	Peripheral incision	15
	Upper abdominal incision	15
	Intrathoracic incision	24
Surgery duration	<2 h	0
	2–3 h	16
	>3 h	23
Emergency procedure	No	0
	Yes	8
**Risk**	**ARISCAT Score**	
Low	<26	
Intermediate	26–44	
High	≥45	

**Table 2:** STOP-BANG for obstructive sleep apnea [13].

STOP	
Snoring	Do you **S**NORE loudly (louder than talking or loud enough to be heard through a closed door)?
Tiredness	Do you often feel **T**IRED, fatigued, or sleepy during daytime?
Observed apnea	Has anyone **O**BSERVED you stop breathing during your sleep?
Pressure	Do you have or are you being treated for high blood **P**RESSURE?
**BANG**	
BMI	**B**MI more than 35 kg/m^2^?
Age	**A**GE over 50 years old?
Neck	**N**ECK circumference > 16 inches (40 cm)
Gender	**G**ENDER: male?
High risk for OSA	5–8 positive responses
Medium risk for OSA	3–4 positive responses
Low risk for OSA	0–2 positive responses
